# Integrated Multi-Omics Analysis to Investigate the Molecular Mechanisms Underlying the Response of *Auricularia heimuer* to High-Temperature Stress

**DOI:** 10.3390/jof11030167

**Published:** 2025-02-20

**Authors:** Fang Lu, Xin Sun, Xiaodong Dai, Piqi Zhang, Yinpeng Ma, Yafei Xu, Lei Wang, Jiechi Zhang

**Affiliations:** 1 Institute of Microbiology, Heilongjiang Academy of Sciences, Harbin 150010, China; fanglulucien@163.com (F.L.); heiweihlj@126.com (X.D.); zhangpiqi@126.com (P.Z.); myp19870315@163.com (Y.M.); 17638066573@163.com (Y.X.); 2 Department of Biotechnology, Institute of Advanced Technology, Heilongjiang Academy of Sciences, Harbin 150001, China; sunxin@cau.edu.cn

**Keywords:** *Auricularia heimuer*, high-temperature stress, transcriptome, metabolome

## Abstract

High-temperature stress is a key factor that reduces the yields of edible fungi. *Auricularia heimuer* (*A. heimuer*) is a nutrient-rich edible fungus that is widely cultivated in China. In this study, we analyzed the physiological, transcriptomic, and metabolomic results of *A. heimuer* (variety “Hei29”) under high-temperature stress. Our findings revealed that high temperatures (30 °C and 35 °C) significantly reduced hyphal growth, increased malondialdehyde content and antioxidant enzyme activity, and enhanced the accumulation of secondary metabolites, such as phenolic compounds and flavonoids. A total of 15 candidate genes potentially responsive to high-temperature stress were identified through transcriptomic analysis, including those involved in regulating antioxidant defense, heat shock response, sugar metabolism, amino acid metabolism, and accumulating secondary metabolites. Metabolomic analysis identified three candidate metabolites potentially responsive to high-temperature stress, including kinetin, flavonoids, and caffeic acid, as well as several metabolic pathways, including nucleotide metabolism, ABC transporters, and cofactor biosynthesis. These mechanisms help mitigate oxidative damage to cellular structures and energy deficits caused by elevated temperatures, enabling the fungus to maintain cellular stability, metabolic function, and growth under heat stress. This study is the first to explore the molecular mechanism of *A. heimuer* in response to high-temperature stress. The results provide valuable insights into the molecular mechanisms of heat stress tolerance in *A. heimuer*, highlighting potential targets for developing heat-tolerant strains for industrial application.

## 1. Introduction

High-temperature stress poses a significant challenge to edible fungi [1], both in natural and artificial cultivation settings, and is a significant factor contributing to yield declines [2]. High temperatures during critical growth stages may lead to mycelial death, contamination of the substrate, and an increase in malformed mushrooms [3], thus affecting the yield. In 2023, Guo et al. [4] found that the optimal growth temperature for *Lentinula edodes* mycelium is 24–27 °C. Heat stress appears to be the significant abiotic constraint inhibiting mycelium growth, disease resistance, and fruiting body development, thereby seriously reducing fruiting body productivity and the quality of *L. edodes* [5]. In 2024, a study by Hu et al. [6] proved that high-temperature stress can affect the production of edible fungi. The study indicated that high-temperature stress (36 °C) inhibits mycelial growth, promotes cell death, and increases the susceptibility of *Pleurotus ostreatus* to infections by *Trichoderma asperellum*. To address this challenge, extensive research has been conducted to unravel the physiological, biochemical, and molecular mechanisms underlying the responses of edible fungi to high-temperature stress. These studies have identified several key factors involved in temperature stress tolerance, including heat shock proteins (HSPs), antioxidant enzymes, and specific signaling pathways. For example, Wang et al. [7] found that heat shock protein 40 (LeDnaJ) in *Lentinula edodes* plays a crucial role in stress resistance and indole-3-acetic acid biosynthesis. Yang et al. [8] discovered that the expression of the catalase gene (*VvCAT1*) from *Volvariella volvacea* in *Escherichia coli* results in recombinant strains exhibiting enhanced heat and cold tolerance. In 2024, Xie et al. [9] found that mTOR and Ca^2+^ signaling pathways were activated in *G. frondosa* under high temperatures and may be involved in heat stress signaling transduction. However, previous studies have only conducted preliminary investigations into the genes and proteins that may be involved in the temperature stress response of edible fungi, with little understanding of the molecular pathways and metabolic adaptations that contribute to high-temperature stress tolerance. Furthermore, there is almost no research on the key genes and molecular pathways involved in *A. heimuer*’s response to high-temperature stress.

*A. heimuer* [10], commonly known as black fungus, is a widely cultivated edible mushroom belonging to the phylum *Basidiomycota*, class *Agaricomycotina* [11], order *Agaricomycetes*, family *Auriculariales*, and genus *Auricularia* [12]. It typically grows on tree trunks or decaying wood and is characterized by its flattened, flexible fruiting body [13]. Among its varieties, “Hei29” is a notable cultivated strain in China [14]. This mid–late maturing variety is particularly known for its resilience to high and low temperatures, strong adaptability to diverse environmental conditions, and consistently high yields, making it well suited for cultivation in Northeast China and similar ecological regions in spring and autumn. Given its robust stress resistance and exceptional agronomic traits, “Hei29” serves as a valuable model for understanding the stress responses of black fungus and improving other cultivars [15].

Omics analysis, especially multi-omics integrative analysis, has been increasingly applied in edible fungi research in recent years. This technique allows for extensive data mining across various dimensions, resulting in a more comprehensive and in-depth understanding of the molecular mechanisms within organisms [16]. This study investigates the physiological and molecular responses of *A. heimuer* “Hei29” under high-temperature stress. Specifically, it examines changes in antioxidant enzyme activity and antioxidant metabolite levels in mycelia, alongside comprehensive transcriptomic and untargeted metabolomic analyses. In this study, a total of 15 candidate genes and three metabolites involved in the high-temperature stress response of *A. heimuer* were screened; these were mainly involved in heat shock response and antioxidant defense metabolic pathways. *A. heimuer* may respond to external high-temperature stress through the activation of heat shock proteins, antioxidant enzyme activity mechanisms, and signaling pathways; specifically, the candidate genes included those encoding HSPs, such as HSP70 and HSP90, antioxidant enzymes, such as Glutathione S-transferase(GST), and transcription factors regulating stress responses, such as C_2_H_2_. The metabolic pathways were enriched in secondary metabolite biosynthesis, including flavonoid and phenolic acid pathways, which contribute to antioxidant activity; additionally, the activation of key signaling pathways, such as the MAPK signaling pathway, played a crucial role in regulating redox homeostasis. These findings suggest that *A. heimuer* responds to external high-temperature stress by adjusting heat shock protein expression, activating antioxidant enzyme systems, increasing antioxidant activity, and modulating signaling pathways to maintain cellular stability and mitigate oxidative damage.

This research further explores temperature signal transduction pathways, gene expression regulation, and metabolic adjustments, providing insights into the adaptive strategies employed by *A. heimuer* in response to temperature fluctuations. The findings contribute to a broader understanding of the mechanisms underlying fungal temperature tolerance and offer valuable guidance for improving the cultivation and breeding of stress-resilient edible fungi.

## 2. Materials and Methods

### 2.1. Fungi Materials and Treatment

The test strain, “Hei29 (*A. heimuer*)”, was provided by the Culture Collection Center of the Microbiology Research Institute, Heilongjiang Academy of Sciences. A continuous stress cultivation method was employed. Strains were uniformly germinated at 26 °C for 2 days and then transferred to 25 °C, 30 °C, and 35 °C for continuous stress cultivation. The medium was prepared by boiling 200 g of diced potatoes in distilled water, adding 20 g of glucose (Aobox, Beijing, China) and 18 g of agar powder (Aobox, Beijing, China), and then adding distilled water to reach a volume of 1000 mL, followed by autoclaving at 121 °C for 30 min. After solidification, the medium was covered with cellulose acetate membrane (Solarbio, Beijing, China) to facilitate sampling. Fungal blocks with a diameter of 11 mm were cut from the active growing edge of the mycelium using a sterile perforator (Jinze, Zhengzhou, China) and then inoculated into the cellulose acetate membrane. Mycelium samples (~0.1 g) were collected from each treatment group at the 9th day for the physiological analysis and omics analysis, with three biological replicates collected at each time point.

### 2.2. Physiological Analysis

Antioxidant enzyme systems and secondary metabolites, such as flavonoids, were quantified using the microplate method (commercial kit information is shown in Table 1). Phosphate-buffered saline (PBS, pH = 7.4) was used for superoxide dismutase (SOD) activity, catalase (CAT) activity, laccase activity, GST activity, glutathione (GSH) content, malondialdehyde (MDA) content and reducing sugar content. The 0.1 g wet sample of mycelium was scraped from the cellulose acetate membrane using a sterile scalpel, mixed with quartz sand, and suspended in 1 mL PBS buffer for grinding before further analysis. The SOD activity was determined using the Water-Soluble Tetrazolium-8 (WST-8) method, following the protocol described by Tang et al. [17], with absorbance measured at 450 nm. CAT activity was measured according to the method of Gao et al. [18], with detection at 520 nm. GSH content was quantified using the 5,5′-Dithiobis(2-Nitrobenzoic Acid) (DTNB) method as described by Alpert et al. [19], with absorbance recorded at 412 nm.

GST activity was assessed using the 1-Chloro-2,4-Dinitrobenzene (CDNB) conjugation assay, following Du et al. [20], with absorbance measured at 340 nm. MDA content was determined using the thio-barbituric acid (TBA) method, based on the protocol of Xu et al. [21], with absorbance measured at 532 nm and corrected using values at 450 nm and 600 nm. Reducing sugar content was quantified using the dinitro-salicylic acid (DNS) method, according to Brunton et al. [22], with absorbance measured at 540 nm.

The 0.1 g wet sample of mycelium was scraped from the cellulose acetate membrane using a sterile scalpel, mixed with quartz sand, and suspended in 1 mL of 60% ethanol for grinding and then subjected to ultrasound at 100 W for 30 min at 60 °C (Shumei, Kunshan, China) before further analysis. Total phenolic content was determined based on the phosphotungstic–phosphomolybdic acid reduction method, following Wang et al. [23], with detection at 760 nm. Flavonoid content was measured using the aluminum chloride colorimetric method, as described by Wu et al. [24], with absorbance recorded at either 470 nm or 502 nm.

### 2.3. Transcriptome Analysis

For RNA extraction, 0.1 g of mycelium samples cultured at 25 °C, 30 °C, and 35 °C for nine days were collected in three biological replicates. The samples were ground into a fine powder under liquid nitrogen and stored at −80 °C until further use. Total RNA was extracted from mycelium samples using the Quick RNA Isolation Kit (Huayueyang, Beijing, China) [15]. RNA concentration and integrity were determined using a nucleic acid protein detector and agarose gel electrophoresis [25]. RNA-seq transcriptome analysis was conducted using the Majorbio Cloud Platform (https://cloud.majorbio.com/, accessed on 1 May 2024). Library construction and sequencing were performed by Shanghai Majorbio Bio-pharm Biotechnology Co., Ltd. (Shanghai, China), following Illumina’s standard protocol [26].

Gene expression levels were quantified using the transcripts per million (TPM) method via RSEM. Differentially expressed genes (DEGs) were identified using DESeq2 or DEGseq, with thresholds of |log2FC| ≥ 1 and FDR < 0.05 (DESeq2) or FDR < 0.001 (DEGseq). Functional enrichment analysis of DEGs was conducted using GO (Gene Ontology) and KEGG (Kyoto Encyclopedia of Genes and Genomes) databases. Enriched GO terms and pathways were identified at a Bonferroni-corrected *p*-value of <0.05, utilizing Goatools 1.4.10 and Python 3.10 scipy for GO and KEGG analysis, respectively.

### 2.4. Real-Time–Quantitative PCR (RT–qPCR) Analysis

The mycelia of Hei29 cultured on solid media for 9 days at 25 °C, 30 °C, and 35 °C were collected, and RNA was extracted. Subsequently, reverse transcription was performed, followed by quantitative real-time–PCR (qRT–PCR) analysis. The extracted RNA was reverse-transcribed into cDNA using the PrimeScript FAST RT Reagent Kit (TAKARA BIO Inc., Kusatsu, Shiga, Japan) with gDNA Eraser. RT–qPCR was performed using the SYBR Green QuantiTect RT–PCR kit (LABLEAD, Beijing, China)under the following cycling conditions: initial denaturation at 95 °C for 10 s, followed by annealing at 60 °C for 20 s and extension at 72 °C for 30 s, repeated for 40 cycles. Gene expression levels were normalized to the expression of β-tubulin (βTUB) [27], and relative gene expression was calculated using the 2^−∆∆CT^ method. The primer sequences used for RT–qPCR are provided in Table S1.

### 2.5. Metabolome Analysis

For metabolomic analysis, 0.1 g of mycelium samples cultured at 25 °C, 30 °C, and 35 °C for nine days were collected. The samples were scraped from the cellophane membrane, immediately frozen in liquid nitrogen, and stored at −80 °C until extraction. Prior to analysis, the frozen samples were ground into a fine powder under liquid nitrogen and subjected to metabolite extraction using an acetonitrile/water system. Non-targeted metabolomic detection was performed using Agilent 8890-7000D and Agilent 8890-5977B GC-MS (Agilent Technologies, Santa Clara, CA, USA) instruments, as well as SCIEX QTRAP (AB SCIEX, Framingham, MA, USA) and Thermo Fisher Q-Exective HFX LC-MS (Thermo Fisher Scientific, Waltham, MA, USA) instruments [28]. Raw metabolomic data were converted using Abf Converter and processed in MS–DIAL software (v4.60) for peak data extraction and normalization. Principal component analysis (PCA), orthogonal partial least squares discriminant analysis (OPLS–DA), and volcano plot analyses were performed using the MetaboAnalyst 5.0 online platform (https://www.metaboanalyst.ca/, accessed on 1 May 2024) [29]. Differential metabolites were identified with criteria of fold change > 2, *p*-value < 0.05, and VIP > 1. Metabolic pathways were analyzed using MetaboAnalyst 5.0 and KEGG databases, and a comprehensive metabolic network was constructed based on relationships among potential biomarkers.

## 3. Results

### 3.1. Effects of High-Temperature Stress on Physiological Phenotypes of Hei29

**Figure 1:**
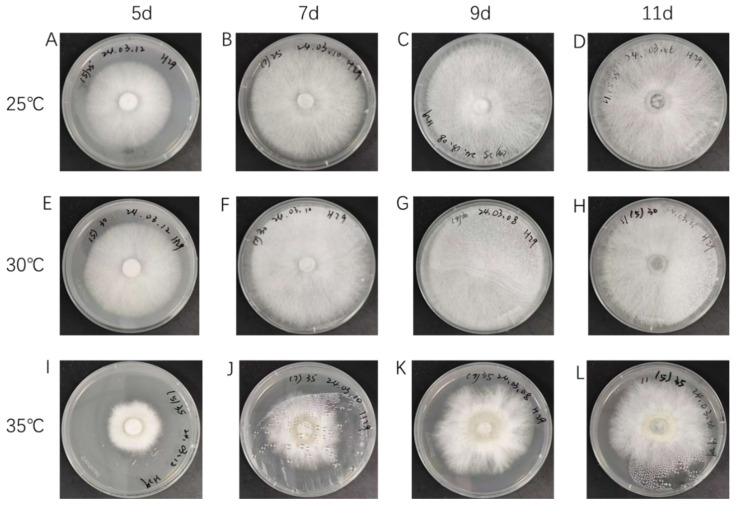
Morphology of Hei29 mycelium at different temperatures and time points. (**A**–**D**) Growth of Hei29 mycelium at 25 °C. (**E**–**H**): Growth of Hei29 mycelium at 30 °C. (**I**–**L**): Growth of Hei29 mycelium at 35 °C.

The growth of Hei29 mycelium exhibited varying characteristics at different temperatures. At 25 °C, the mycelium showed normal growth, with a typical appearance and growth pattern; however, the mycelium grew vigorously at 30 °C, displaying rapid expansion and an abundant, dense network of hyphae. In contrast, at 35 °C, the mycelium experienced a noticeable decrease in growth rate, and pigment deposition was observed, indicating stress responses under this higher temperature (Figure 1). It can also be seen from Figure 1 that the difference in Hei29 mycelia growth under different temperature treatments was most obvious on the 7th and 9th day. Combined with the results of the pre-experiment, the mycelium under different temperature treatments showed the most significant differences in physiological indicators on the ninth day (Figure S1). Therefore, the ninth day was selected as the sampling time for the temperature stress treatment for subsequent experimental analysis.

**Figure 2:**
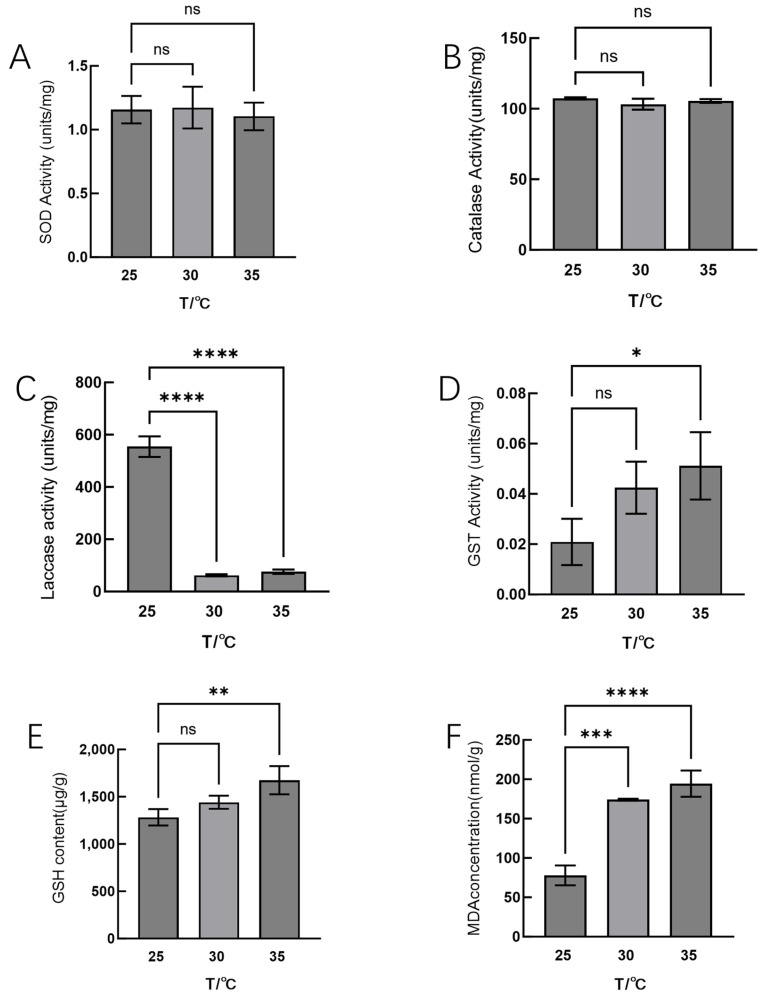
Detection of physiological indicators of Hei29 mycelia under different temperature stresses. (**A**) SOD activity, (**B**) CAT activity, (**C**) laccase activity, (**D**) GST activity, (**E**) GSH content, and (**F**) MDA concentration in Hei29 mycelia exposed to different temperature treatments for nine days. ns indicates *p* > 0.05 (no statistical significance), * *p* ≤ 0.05, ** *p* ≤ 0.01, *** *p* ≤ 0.001, **** *p* ≤ 0.0001.

Subsequently, we examined the activities of antioxidant enzymes in Hei29 mycelium under different temperature treatments (Figure 2). SOD activity showed no significant differences among 25 °C, 30 °C, and 35 °C. (Figure 2A). The changes in CAT activity in Hei29 mycelium under different temperatures are similar to those of SOD, with no significant differences (Figure 2B). GST activity increased at 30 °C and 35 °C compared with the control, but a significant difference was only observed at 35 °C (Figure 2C).

Laccase activity varied significantly at different temperatures. Laccase activity decreased significantly at 30 °C and 35 °C compared with 25 °C, and there was no significant difference in laccase activity between 30 °C and 35 °C (Figure 2D).

Fluctuations in temperature may impact the levels of intracellular oxidative stress, and measurement of the biomarkers of oxidative damage can provide direct evidence of the extent of cellular damage. We examined the indicators of oxidative damage. GSH levels increased significantly at both 30 °C and 35 °C compared with 25 °C, with the increase being more pronounced at 35 °C; additionally, GSH levels remained elevated at 35 °C, with the differences being significant (Figure 2E). MDA content also significantly increased at both 30 °C and 35 °C compared with 25 °C; the increase was more pronounced at 35 °C (Figure 2F).

**Figure 3:**
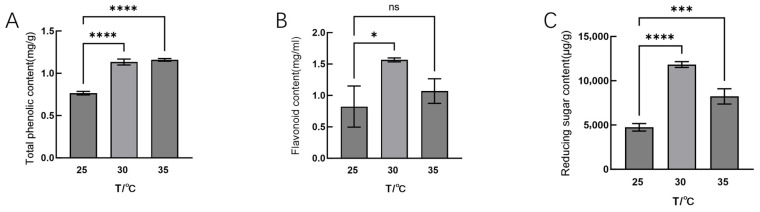
Content of metabolites in Hei29 mycelium under different temperature stresses. (**A**) Total phenolic content under different temperature treatments. (**B**) Flavonoid content under different temperature treatments. (**C**) Reducing sugar content under different temperature treatments. Treatments were conducted for nine days. ns indicates *p* > 0.05 (no statistical significance), * *p* ≤ 0.05, *** *p* ≤ 0.001, **** *p* ≤ 0.0001.

In addition to the antioxidant system, changes in mycelial carbohydrate metabolism and secondary metabolites also play a crucial role in mycelium’s adaptability and growth under high-temperature stress; therefore, we examined the contents of metabolism and secondary metabolites in Hei29 mycelia under different temperature stresses. As can be seen from the results, total phenol content increased significantly at both 30 °C and 35 °C compared with 25 °C, with the highest levels observed at 35 °C (Figure 3A). Flavonoid content significantly increased at 30 °C, with a slight increase at 35 °C (Figure 3B). The reducing sugar content significantly increased at both 30 °C and 35 °C, with a higher accumulation at 30 °C; however, at 35 °C, the reducing sugar content slightly decreased compared with 30 °C (Figure 3C).

### 3.2. Transcriptomic Analysis Under High-Temperature Stress

**Figure 4:**
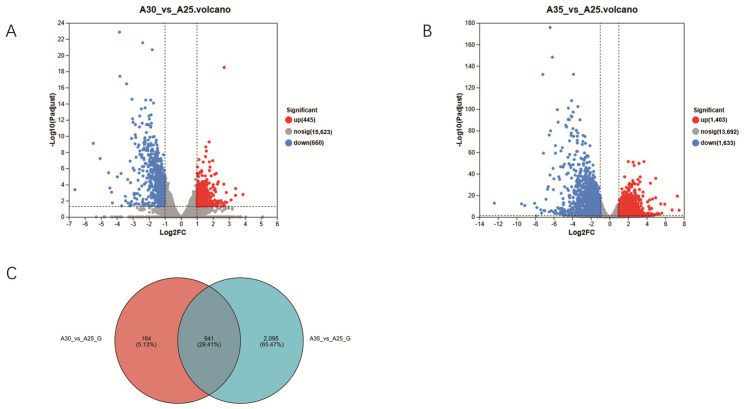
Statistical analysis of differentially expressed genes in Hei29 mycelium under different temperature stresses. (**A**) Differentially expressed genes in Hei29 mycelium under 30 °C stress. (**B**) Differentially expressed genes in Hei29 mycelium under 35 °C stress. (**C**) Venn diagram of genes that are differentially expressed at 30 °C and 35 °C. Blue represents downregulated genes while red represents upregulated genes. Samples cultured at 25 °C were used as the control group.

To further explore the molecular mechanisms of Hei29 in response to heat stress, transcriptomics was used to screen for genes potentially involved in temperature stress response. Samples cultured at 25 °C were used as the control group. As the results show, 1105 DEGs were identified under the 30 °C condition compared with the control condition (25 °C), of which 445 genes were upregulated and 660 genes were downregulated (Figure 4A). A more extensive set of 3036 DEGs was detected under the 35 °C condition compared with the control, with 1403 genes upregulated and 1633 genes downregulated (Figure 4B). A total of 941 genes were differentially expressed under both 30 °C and 35 °C conditions, accounting for 29.41% of the differentially expressed genes. These include regulatory genes associated with antioxidant enzymes (Figure 4C).

**Figure 5:**
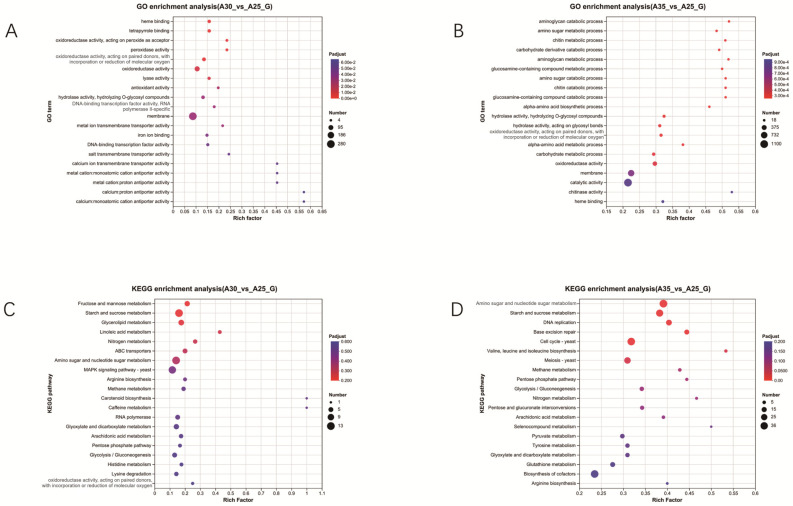
Analysis of GO terms and KEGG pathway enrichment in Hei29 under different temperature stresses. (**A**) GO analysis of differentially expressed genes (DEGs) in Hei29 mycelium under 30 °C stress. (**B**) GO analysis of DEGs in Hei29 mycelium under 35 °C stress. (**C**) KEGG analysis of DEGs in Hei29 mycelium under 30 °C stress. (**D**) KEGG analysis of DEGs in Hei29 mycelium under 35 °C stress. The bubble size represents the number of genes enriched in each pathway, while the color gradient indicates the significance level, with a redder color indicating a smaller adjusted *p* adjust value and higher confidence in the enrichment. Samples cultured at 25 °C were used as the control group.

GO enrichment analysis at 30 °C revealed that enriched functional categories are primarily associated with membrane-associated functions and antioxidant activities; these include “membrane transport activities” and “oxidoreductase activity”. This suggests that the mycelium increases membrane stability and antioxidant capacity at 30 °C to cope with elevated temperatures (Figure 5A). KEGG pathway enrichment analysis at 30 °C revealed that enriched metabolic pathways include those involved in fructose and mannose metabolism, starch and sucrose metabolism, amino sugar and nucleotide sugar metabolism, and the MAPK signaling pathway yeast. This suggests that the mycelium activates signaling and metabolic regulation mechanisms at 30 °C to adapt to temperature changes (Figure 5C).

GO enrichment analysis at 35 °C revealed that enriched functional categories are mainly associated with carbohydrate metabolic processes, oxidoreductase activity, membrane transport, and catalytic activity. This indicates that the mycelium enhances catalytic activities and membrane transport capabilities at 35 °C to adapt to high temperatures (Figure 5B). KEGG enrichment analysis at 35 °C revealed that enriched metabolic pathways include those involved in amino sugar and nucleotide sugar metabolism, starch and sucrose metabolism, DNA replication, base excision repair, and cell cycle (Figure 5D).

### 3.3. RT–PCR Verification of DEGs

RT–PCR was employed to validate the expression levels of 15 key genes involved in the heat stress response of Hei29. The selection of key genes was based on differential expression analysis, focusing on genes associated with redox reactions and cellular metabolism. The reference gene used for normalization was *TUB-1α*.

**Figure 6:**
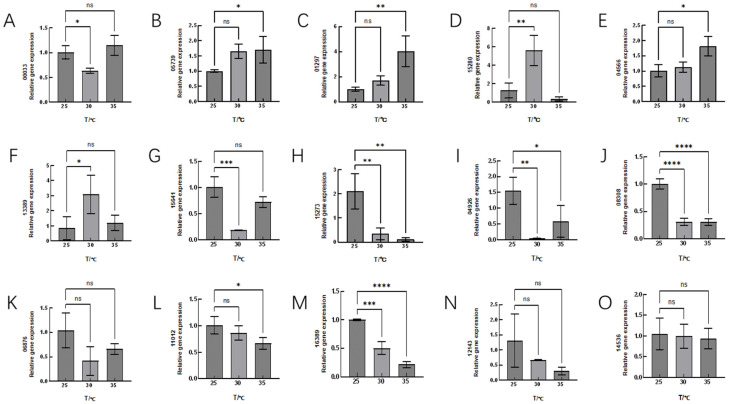
Expression levels of 15 candidate genes expressed under high-temperature stress. (**A**) *Gene_00033*, (**B**) *Gene_05739*, (**C**) *Gene_01297*, (**D**) *Gene_15280*, (**E**) *Gene_04566*, (**F**) *Gene_13389*, (**G**) *Gene_15641*, (**H**) *Gene_15273*, (**I**) *Gene_04926*, (**J**) *Gene_08308*, (**K**) *Gene_06876*, (**L**) *Gene_11012*, (**M**) *Gene_16389*, (**N**) *Gene_12143*, and (**O**) *Gene_14536.* The expression levels of these genes were analyzed by quantitative real-time–PCR (qRT–PCR) under heat stress at different temperatures over a 9-day period. The data are presented as relative expression levels compared with the control group. ns indicates *p* > 0.05 (no statistical significance), * *p* ≤ 0.05, ** *p* ≤ 0.01, *** *p* ≤ 0.001, **** *p* ≤ 0.0001.

The qRT–PCR results indicate varied expression patterns of key genes involved in stress response and metabolism. Genes such as GCLC (*Gene_05739*), the isoflavone reductase family (*Gene_01297*), GST (*Gene_15641*), SOD2 (*Gene_08308*), and copper oxidase (*Gene_10345*) were upregulated, suggesting activation of antioxidant defense and metal ion regulation; in contrast, genes such as tyrosinase (*Gene_15280* and *Gene_13389*) and laccase (*Gene_07657*) were downregulated, indicating potential suppression of certain oxidative stress responses. The expression patterns of these genes were generally consistent with the trends observed in the transcriptomic analysis presented in Figure 6.

### 3.4. Metabolomic Analysis of Auricularia Under High-Temperature Stress

**Figure 7:**
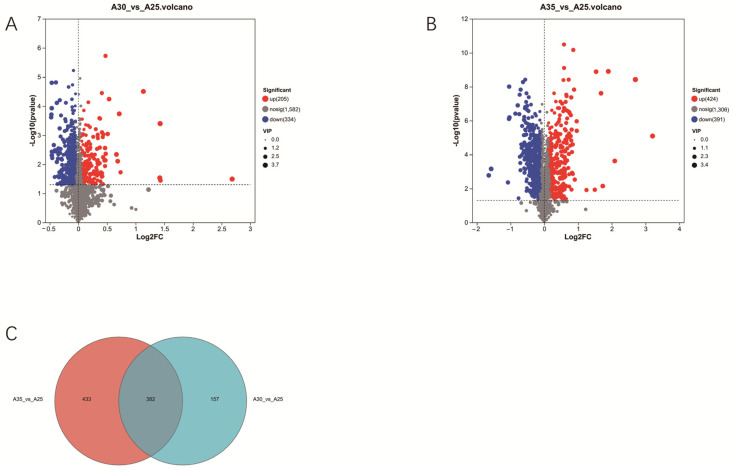
Statistical analysis of differentially expressed metabolites in Hei29 mycelium under different temperature stresses. (**A**) Differentially expressed metabolites in Hei29 mycelium under 30 °C stress. (**B**) Differentially expressed metabolites in Hei29 mycelium under 35 °C stress. (**C**) The number of differentially expressed metabolites in Hei29 mycelium under different temperature stresses. Blue represents downregulated metabolites while red represents upregulated metabolites. Samples cultured at 25 °C were used as the control group.

Volcano plots of differential metabolite expression reveal distinct shifts in metabolite regulation under different temperature conditions. Samples cultured at 25 °C were used as the control group. At 30 °C, 205 metabolites were significantly upregulated, while 334 were significantly downregulated (Figure 7A). At 35 °C, 424 metabolites were significantly upregulated, while 391 were significantly downregulated (Figure 7B). The smaller number of significantly altered metabolites at 30 °C suggests a more moderate metabolic response than the more extensive changes observed at 35 °C. A total of 382 metabolites were identified based on the comparison of differentially expressed metabolites. It was observed that metabolites were significantly upregulated under the 30 °C and 35 °C conditions; these included auxins, flavonoids, and phenolic acids (Figure 7C).

**Figure 8:**
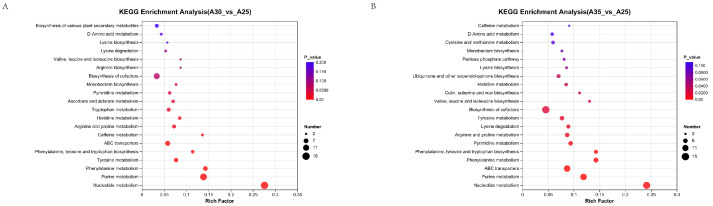
KEGG pathway enrichment analysis of metabolites differentially expressed under heat stress. (**A**) KEGG pathway analysis of differentially expressed metabolites in Hei29 mycelium under 30 °C stress. (**B**) KEGG pathway analysis of differentially expressed metabolites in Hei29 mycelium under 35 °C stress. The bubble size represents the number of metabolites enriched in each pathway, while the color gradient indicates the significance level, with a redder color indicating a smaller adjusted *p* adjust value and higher confidence in the enrichment. Samples cultured at 25 °C were used as the control group.

At 30 °C, KEGG pathway enrichment analysis of the metabolome revealed that pathways associated with nucleotide metabolism, purine metabolism, ABC transporters, biosynthesis of cofactors, and tyrosine metabolism were significantly enriched in comparison to the control group (Figure 8A).

At 35 °C, KEGG pathway enrichment analysis of the metabolome revealed that the pathways associated with cofactor biosynthesis, ABC transporters, nucleotide metabolism, purine metabolism, ubiquinone and other terpenoid-quinone biosynthesis, and arginine and proline metabolism were significantly enriched in comparison to the control group (Figure 8B).

By integrating transcriptomic and metabolomic data and filtering for significant pathways (*p* < 0.05), distinct KEGG pathways were enriched for 30 °C stress and 35 °C stress. The pathways enriched at 30 °C included those involved in glutamate metabolism, phenolic acid metabolism, and biosynthesis of cofactors; the pathway enriched at 35 °C was that of ABC transporters. These pathways highlight the differential metabolic and molecular responses of Hei29 under moderate and severe temperature stresses.

## 4. Discussion

High-temperature stress significantly impacts the yield and quality of edible fungi [30], particularly *A. heimuer.* Investigating the potential mechanisms underlying the heat stress response in *A. heimuer* and identifying the key genes [31] and metabolic pathways [32] involved in this response are of great importance for enhancing the heat tolerance of this mushroom. In this study, we comprehensively evaluated the physiological responses of *A. heimuer* mycelium under varying temperature conditions (25 °C, 30 °C, and 35 °C) by analyzing antioxidant enzyme activities, oxidative stress markers, and transcriptome and metabolite levels. We identified candidate genes that may respond to high-temperature stress, including antioxidant enzymes, heat shock proteins, and genes associated with flavonoid biosynthesis. We also identified some metabolic pathways that may be involved in high-temperature stress, such as the phenolic acid metabolism pathway. These pathways play a significant role in the heat stress response of *A. heimuer* and contribute to its adaptive mechanisms. These findings provide valuable insights into the molecular mechanisms underlying the response of Hei29 mycelium to high-temperature stresses.

### 4.1. Physiological Changes in Hei29 Under High-Temperature Stress

Mycelial growth is closely correlated to changes in physiological indicators [33]. Changes in mycelial growth are directly linked to the manifestation of oxidative stress under different temperature conditions [34]. As the temperature increases, mycelial growth is gradually inhibited, and the extent of oxidative damage also significantly increases. Changes in physiological indicators, such as antioxidant enzyme activity, MDA content, and secondary metabolites, reflect the oxidative stress response during mycelial growth, further revealing the potential impact of high-temperature stress on mycelial growth.

The levels of MDA, an indicator of lipid peroxidation, varied significantly across temperature conditions. MDA is a product of lipid peroxidation and serves as a marker of cell membrane damage. In 2022, Worasitikulya Taratima et al. [35] found that, after heat stress treatment in rice, both MDA levels and the proportion of electrolyte leakage significantly increased. The results of this study show that MDA content was significantly higher at both 30 °C and 35 °C compared with 25 °C, with a more pronounced increase at 35 °C. This suggests that oxidative damage in the mycelia of *A heimuer* intensifies under high-temperature stress, especially at 35 °C, where membrane damage becomes more evident, indicating that elevated temperatures may lead to stronger lipid peroxidation reactions.

Edible mushrooms, when subjected to oxidative damage, can mitigate cellular oxidative stress by increasing the activity of antioxidant enzymes, such as POD, SOD, and CAT, or by enhancing the levels of antioxidant compounds to reduce oxidative damage to the cells [36]. GSH is a type of antioxidant compound. In 2016, Dogan et al. [37] identified GSH as a key component of the antioxidant system of eight edible mushroom species; GSH plays a crucial role in the cells of these mushrooms, particularly in combating oxidative stress and protecting cells from damage by free radicals. In the current study, GSH levels significantly increased at both 30 °C and 35 °C compared with 25 °C, with the most pronounced increase being observed at 35 °C. These results suggest that elevated temperatures trigger the upregulation of GSH as a protective mechanism against oxidative stress.

The activities of SOD and CAT did not show significant variations under temperature stress, indicating that *A heimuer* does not primarily rely on these enzymes to regulate oxidative stress. These observations are in accordance with the study conducted by Hu et al. [6] (2022), which indicated that *Pleurotus ostreatus* does not primarily rely on SOD or CAT for adaptation to heat stress. Instead, the study suggested that metabolic reprogramming induced by salicylic acid may play a pivotal role in the thermotolerance of *P. ostreatus*; in contrast, *A. heimuer* may employ alternative antioxidant mechanisms or stress response pathways, such as the accumulation of antioxidant active compounds and secondary metabolites, to cope with high-temperature stress. In this study, the activity of GST significantly increased under high-temperature stress. GST plays a vital role in detoxification and antioxidant processes. This finding aligns with the conclusions of Li et al. (2024) [38], who suggested that GST plays a role in mitigating oxidative damage and enhancing heat tolerance in *Pleurotus ostreatus* through the SIRT2-mediated deacetylation. Similarly, in Hei29, the elevated GST activity at 35 °C indicates that GST may help the mycelium cope with high-temperature stress by alleviating oxidative damage, supporting its role as an important antioxidant defense mechanism in response to heat stress.

Laccase activity significantly decreased at 30 °C and 35 °C compared with 25 °C. This decline in laccase activity under thermal stress suggests a diminished capacity for oxidative stress response and lignocellulose degradation. However, since lignocellulose was not a component of the culture medium, the decrease in laccase activity is more likely attributed to thermal stress rather than reduced lignocellulose degradation ability [39]. Similar results were obtained by Wang et al. (2019) [40] in *Lentinus edodes*, where high temperatures led to a significant decrease in laccase activity and reduced gene expression of laccase isoenzymes, likely due to thermal damage and impaired metabolic pathways. This suggests that, under heat stress, *A. heimuer* may prioritize energy allocation toward maintaining cell integrity and stress tolerance rather than lignocellulose degradation, leading to a slower growth rate and reduced laccase activity [41].

The alterations in secondary metabolites reflect the mycelium’s response to stress through the modulation of metabolic pathways; therefore, monitoring these indicators provides a comprehensive understanding of the impact of high-temperature stress on mycelial growth.

Reducing sugar content significantly increased at both 30 °C and 35 °C, with a higher accumulation at 30 °C; however, the reducing sugar content slightly decreased at 35 °C compared with 30 °C. This suggests that the mycelium accumulates reducing sugars as an adaptive response to high-temperature stress, likely to support energy metabolism and osmotic regulation. The slight decrease at 35 °C could indicate a metabolic shift or a stress-induced disruption of carbohydrate metabolism under more extreme conditions. Together with the decreased laccase activity, these results suggest that Hei29 might compensate for lignin degradation deficiency by regulating carbohydrate metabolism to maintain its growth and physiological balance under high-temperature stress.

Phenolic compounds are crucial in protecting cells from oxidative damage, acting as scavengers of reactive oxygen species (ROS) [42]. In 2022, Zhao et al. [43] found that the synthesis of phenolic compounds enhanced the ability to scavenge ROS, thereby delaying the deterioration of rice grains. The total phenol content was higher at both 30 °C and 35 °C compared with 25 °C, with the highest levels observed at 35 °C. This suggests that, at 35 °C, Hei29 may accumulate more phenolic compounds as an adaptive mechanism against higher temperature stress. The increased phenol content could help the mycelium enhance its antioxidant defense, thereby protecting cells from oxidative damage caused by elevated temperatures.

Flavonoids, which are known for their antioxidant properties, are likely to help the mycelium mitigate oxidative damage caused by elevated temperatures. In 2024, Liu et al. [44] discovered that the SmEGY3-SmCSD1 module can promote the flavonoid biosynthesis mediated by SmF3H, enhancing the heat tolerance of eggplants. The flavonoid content significantly increased at 30 °C, with a slight increase at 35 °C. This suggests that Hei29 may enhance its flavonoid production as part of its adaptive response to moderate heat stress at 30 °C.

### 4.2. Transcriptomic Analysis Under Different Temperature Treatments

The findings from the GO and KEGG pathway enrichment analyses at 30 °C suggest that Hei29 mycelium responds to moderate temperature stress through a complex regulatory network involving membrane stabilization, antioxidant defense, and metabolic and signaling pathways. The enrichment of membrane transport functions suggests that the mycelium is enhancing the efficiency of nutrient and ion exchange across cell membranes. This helps maintain cellular homeostasis under stress conditions, enabling the mycelium to adapt to altered environmental conditions [45]. The enrichment of oxidoreductase activity indicates that the mycelium is upregulating antioxidant enzymes to counteract the increase in ROS produced under heat stress. This suggests a cellular strategy to mitigate oxidative damage and preserve cell integrity during temperature fluctuations. The enrichment of the starch and sucrose metabolism pathway suggests that the mycelium may be modifying its starch and sucrose storage and utilization in response to elevated temperatures. This could be a strategy to ensure energy availability during stressful conditions, enabling the mycelium to maintain its growth and metabolic processes. Analysis of reducing sugar content showed that the accumulation of reducing sugars in the mycelium significantly increased at both 30 °C and 35 °C, consistent with KEGG pathway enrichment, indicating an increase in energy supply during this process. Enrichment of the amino sugar and nucleotide sugar metabolism pathways indicates that the mycelium is regulating the metabolism of amino sugars and nucleotide sugars, which are important for cell wall biosynthesis and signaling. This could help the mycelium adapt to the altered temperature by supporting structural integrity and cellular communication. Dogan et al. [37] found that heat stress activates the cell wall integrity (CWI)–mitogen-activated protein kinase (MAPK) signaling cascade, promoting the expression of heat shock proteins (HSPs). At the same time, heat stress inhibits protein synthesis, induces the production of ROS, and triggers oxidative stress responses, which also stimulate the expression of HSPs. Enrichment of the MAPK signaling pathway suggests that the mycelium is activating a stress response cascade, which is critical for mediating cellular responses to environmental changes, such as temperature stress. MAPK pathways regulate processes like gene expression, cell differentiation, and stress tolerance, and their activation at 30 °C suggests an adaptive mechanism to mitigate the effects of heat stress.

Hei29 employs a multifaceted strategy to cope with moderate heat stress at 30 °C. The mycelium enhances membrane stability and activates antioxidant defenses to protect against oxidative damage; additionally, it modulates its metabolic processes, increasing the breakdown of sugars for energy, adjusting carbohydrate storage, and regulating cell wall components to maintain cellular integrity. The activation of the MAPK signaling pathway suggests that the mycelium is also engaging in a complex stress response mechanism, coordinating molecular and metabolic changes to adapt to the temperature shift. The enrichment of carbohydrate-metabolism-related functions suggests that the mycelium is actively adjusting its energy metabolism to cope with the stress induced by high temperatures. This could involve the enhanced breakdown of stored carbohydrates (like starch and sucrose) to generate energy, which is crucial for maintaining cell functions and supporting the stress response. The presence of oxidoreductase activity in the enriched categories indicates that the mycelium is upregulating enzymes involved in antioxidant defense. These enzymes help neutralize ROS generated during heat stress, protecting the mycelium from oxidative damage and maintaining cellular integrity. The enrichment of membrane and catalytic activities suggests that the mycelium is enhancing its membrane transport systems and enzymatic functions. Similar to the 30 °C condition, the enrichment of the starch and sucrose metabolism pathway at 35 °C suggests that the mycelium continues to regulate carbohydrate storage and utilization; however, at higher temperatures, this may become even more critical for providing energy to support cell survival and growth under stressful conditions.

In this study, transcriptomic analysis identified several candidate genes in Hei29 mycelium that may respond to high-temperature stress. GCLC (*Gene_05739*) [46] participates in the synthesis of glutathione, an important antioxidant that helps protect cells from oxidative damage, which aligns with the increased glutathione levels observed in physiological indicators. The isoflavone reductase family (*Gene_01297*) [47] is involved in the biosynthesis of flavonoids, consistent with the increased flavonoid levels detected in the physiological indicators. GST (*Gene_15641*) encodes GST, an enzyme involved in detoxifying ROS and preventing oxidative stress, which matches the increased GST activity observed. SOD2 (*Gene_08308*) is a key enzyme for detoxifying superoxide free radicals. The unchanged SOD activity might indicate that this enzyme is regulated by multiple genes, helping stabilize expression and repair oxidative damage. Laccase (*Gene_07657*) is an oxidase enzyme; its downregulation observed in this study suggests a decrease in some oxidative stress responses or metabolic changes under heat stress, as confirmed by the assays conducted. The downregulation of HSP70 (*Gene_04926*) is consistent with findings from studies, such as by Ming et al. (2009) [48], who observed decreased HSP70 after prolonged heat stress in the groupers, and Wang et al. (2023) [49], who found that prolonged heat stress reduced the SUMOylation level of TaHsfA1, leading to a shift from an active to an inactive state, thereby weakening or even shutting down heat stress responses (HSRs).

### 4.3. Metabolomic Analysis Under Different Temperature Treatments

The significant upregulation of auxins, flavonoids, and phenolic acids at both 30 °C and 35 °C indicates that these metabolites may play a key role in mycelial response to temperature stress. Kinetin [50], a type of cytokinin plant hormone, is crucial for regulating growth, cell differentiation, and stress responses. The increased levels of kinetin suggest that the mycelium might be activating growth and developmental pathways to adapt to elevated temperatures. Flavonoids are well known for their antioxidant properties. Their upregulation at higher temperatures could indicate that the mycelium is enhancing its antioxidant defenses to neutralize reactive oxygen species (ROS) produced under high-temperature stress. Flavonoids, as secondary metabolites, might also act as protective agents against oxidative damage, contributing to the stabilization of cellular structures and enzymes. Phenolic acids are involved in plant defense mechanisms and are known for their antimicrobial, antioxidant, and UV-protective properties. Their accumulation under heat stress could be a response to combat oxidative damage and to protect the mycelium from thermal injury. Phenolic acids are also involved in strengthening cell walls and modulating signal transduction pathways that help the mycelium adapt to stressful conditions. The increased levels of these metabolites at both 30 °C and 35 °C suggest that the mycelium is producing a variety of antioxidant metabolites to counteract the stress and cope with the heat-induced oxidative damage.

The KEGG pathway enrichment analysis of the metabolome at both 30 °C and 35 °C revealed several significant metabolic pathways compared with the control group.

At 30 °C, the enriched pathways, including those involved in nucleotide metabolism, purine metabolism, ABC transporters, biosynthesis of cofactors, and tyrosine metabolism, suggest that the mycelium is enhancing nucleotide and purine metabolism, which are crucial for energy production and cell function under moderate high-temperature stress. Additionally, the enrichment of ABC transporters indicates active transport mechanisms that may help in the movement of molecules across membranes, essential for maintaining cellular integrity under stress. The involvement of biosynthesis of cofactors and tyrosine metabolism implies a heightened need for metabolic regulation and antioxidant defense at this temperature.

At 35 °C, the pathways enriched include those involved in the biosynthesis of cofactors, ABC transporters, nucleotide metabolism, purine metabolism, ubiquinone and other terpenoid-quinone biosynthesis, and arginine and proline metabolism. These findings suggest an even greater metabolic adaptation to severe heat stress. The addition of ubiquinone and other terpenoid–quinone biosynthesis points to the production of compounds that may help protect cells from oxidative damage. Arginine and proline metabolism enrichment highlight an increased need for amino acids involved in stress responses, osmoregulation, and antioxidation. This indicates that at 35 °C, the mycelium is activating additional pathways for survival under more extreme conditions.

In summary, both temperatures triggered significant metabolic shifts, with 35 °C inducing more comprehensive molecular responses, including enhanced antioxidant mechanisms and metabolic regulation.

## 5. Conclusions

In conclusion, this study reveals key mechanisms underlying high-temperature stress response in Hei29, emphasizing the role of antioxidant enzymes and secondary metabolites in thermal adaptation. The significant increase in GSH, MDA, and GST activity and changes in metabolites, such as total phenols and flavonoids, suggest that oxidative damage to the mycelium is mitigated through enhanced antioxidant defense, particularly by GSH and associated detoxification pathways. A total of 15 candidate genes potentially responsive to high-temperature stress were identified through transcriptomic analysis, including those involved in the regulation of antioxidant defense, high-temperature shock response, sugar metabolism, amino acid metabolism, and the accumulation of secondary metabolites. Metabolomic analysis identified three candidate metabolites potentially responsive to high-temperature stress—kinetin, flavonoids, and caffeic acid—as well as several metabolic pathways, including nucleotide metabolism, purine metabolism, ABC transporters, cofactor biosynthesis, and tyrosine metabolism.

In summary, Hei29 may mitigate cellular oxidative damage and respond to external high-temperature stress by increasing the activity and accumulation of antioxidant enzymes and antioxidants, regulating the expression of high-temperature stress-related genes, and modulating metabolic pathways, such as carbohydrate and phenolic acid metabolism. These findings provide valuable insights into the mechanisms underlying the response of *A. heimuer* to high-temperature stress and are of great significance for breeding stress-resistant strains. The candidate genes, metabolites, and metabolic pathways identified in this study offer new ideas and foundations for molecular breeding of stress-resistant *A. heimuer* and improving its high-temperature tolerance.

## Figures and Tables

**Table 1:** Information on the commercial kits used in this study.

Kit Name	Brand Name	Item No.	Region
Total SOD Activity Assay Kit (WST-8 Method)	Beyotime	S0101S	Shanghai, China
Peroxidase Assay Kit	Beyotime	S0051	Shanghai, China
Reduced Glutathione (GSH) Content Assay Kit	Solarbio	BC1175	Beijing, China
Plant Flavonoid Content Assay Kit	Solarbio	BC1330	Beijing, China
Total Phenol (TP) Content Assay Kit	Solarbio	BC1340	Beijing, China
Glutathione S-Transferase (GST) Activity Assay Kit	Solarbio	BC0350	Beijing, China
Malondialdehyde (MDA) Content Assay Kit	Solarbio	BC0025	Beijing, China
Laccase Activity Assay Kit	Solarbio	BC1630	Beijing, China
Reducing Sugar Content Assay Kit	Solarbio	BC0235	Beijing, China
Quick RNA Isolation Kit	Huayueyang	0416-50GX	Beijing, China
SYBR Green QuantiTect RT–PCR kit	LABLEAD	R0202	Beijing, China
FastKing gDNA Dispelling RT SuperMix kit	TAKARA BIO INC	RR092A	Kusatsu, Shiga, Japan

## Data Availability

The original contributions presented in the study are included in the article/Supplementary Material, further inquiries can be directed to the corresponding authors.

## Supplementary Materials

The following supporting information can be downloaded at: https://www.mdpi.com/article/10.3390/jof11030167/s1, Figure S1: Detection of metabolic indicators in Hei29 mycelia at different cultivation time and temperature. (A) Reducing sugar content and (B) Flavonoid content in Hei29 mycelia cultured at 25, 30, 35 °C collected at 5, 7, 9, and 11 days. Different lowercase letters (a, b, c, d) indicate significant differences between time points (*p* ≤ 0.05); Table S1: Primers used for qRT-PCR analysis.
