# Supplementary material for: Integrated Multi-Omics Analysis to Investigate the Molecular Mechanisms Underlying the Response of Auricularia heimuer to High-Temperature Stress
Source: J Fungi (Basel). 2025 Feb 20;11(3):167. doi: 10.3390/jof11030167 (PMC11943417; doi:10.3390/jof11030167)
Supplement: Supplementary file 1 [file jof-11-00167-s001.zip › jof-3479130-supplementary.pdf]

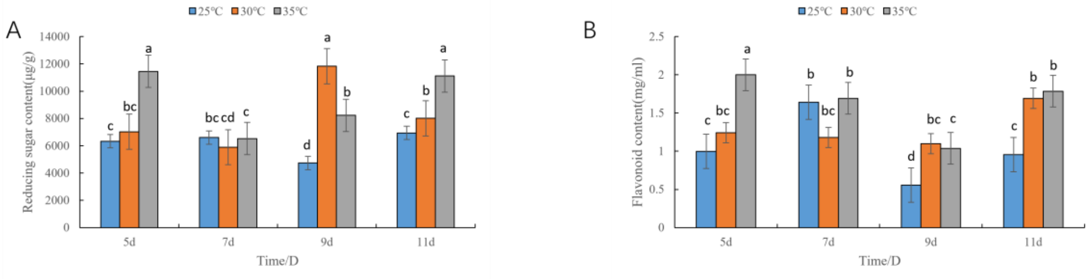

Figure S1. Detection of metabolic indicators in Hei29 mycelia at different cultivation time and temperature

(A) Reducing sugar content and (B) Flavonoid content in Hei29 mycelia cultured at 25, 30, 35 ° C collected at 5, 7, 9, and 11 days. Different lowercase letters (a, b, c, d) indicate significant differences between time points ($p \leq 0.05$).

Table S1. Primers used for qRT-PCR analysis

Gene Name	Forward Primer (5'-3')	Reverse Primer (3'-5')
gene_15641	AGAGCGGTGCCATCGTAG	TTTGGTCCCAAAGGCGCAGGGAAGC
gene_04566	CACTACCCACAGACGACAACC	GCGCCGCGCCAGACGCCGCAACGAA
gene_06876	CCCGACAAGCATCCGAAAC	AGCGTGGTAGGCGGCAAT
gene_05739	TTTCTCCGTGTTCATTGTCTTAT	CGCTTCCTTGCGACTACC
gene_08308	AGAAAGAGTTGAACACGCAGAC	TTCCAGATGGCATTGAGGT
gene_01297	CGCCCAACTACTCTACCAATC	GTCTTTGACGAAATAGCCACC
gene_10345	CGGCAGACTCGGTGTTCG	GCAATCTTGGTTGGTGGG
gene_07657	GTGCTCAATCTTGGTTCGG	GTGTAGTGCCTTCTTCTGGTCT
gene_15280	CATCCACGACACCATCCAT	TACAGCCGCTTCACCTCC
gene_13389	AGTTGCTGGTGGTGTTGTGC	CGCTGCGATACCGATAGATT
gene_15273	GAAAGCATCACGGGCATC	TACGGCACCACAACATCG
gene_01223	ACTTTCCCGTTCACCGACT	ATTGCGATGTCCTCCTTCTT
gene_04926	CTCCGTCTTTTCTCCCACTAC	CGCATACATCTTCGCTGACT
gene_12143	CGGCTTTCATAAAATCAATCG	GGAAGTCTGCTGATGTGGG
gene_14536	TGGGATAAACCTGTGGTGATT	CGCAGTTGATGTTGGGATG
gene_11012	TACGGTCGGACGGGTTAG	CCAGCACGAAGTTGAAGCA

18srRNA	CTGGCTCTGTCAGTGTAG	TCCGATAACGAACGAGAC
gene_07657	GTGCTCAATCTTGGTTCGG	GTGTAGTGCCTTCTTCTGGTCT
gene_00033	GCAGCATTTTATCTCCAACG	AGTTCTCGGTAATCCACTCCA
gene_16398	TTCCTCATCAAGCACCTCAC	GTTCTTCCCCGCCCATAC
β-TUB	TCGCCCAAGGTTTCGGACACT	GACGGTTGAGGTACAGACACGA
EF-2	GATTCTGGCAAGACCACGCTCAC	TGACTGGAATGCTCGCAGACTGA
TUB-1a	GTCACAGTCGCAGCAGCAGAT	CGTCGTCGTCGCTGTTGTCTT
